# Development of a CT imaging phantom of anthromorphic lung using fused deposition modeling 3D printing

**DOI:** 10.1097/MD.0000000000018617

**Published:** 2020-01-03

**Authors:** Dayeong Hong, Sangwook Lee, Guk Bae Kim, Sang Min Lee, Namkug Kim, Joon Beom Seo

**Affiliations:** a Department of Biomedical Engineering, Asan Medical Institute of Convergence Science and Technology, Asan Medical Center, University of Ulsan College of Medicine; b Department of Radiology, University of Ulsan College of Medicine, Asan Medical Center; c Department of Convergence Medicine, University of Ulsan College of Medicine, Asan Medical Center; d ANYMEDI Inc., Seoul, South Korea.

**Keywords:** 3D printing, CT imaging, Hounsfield unit, imaging phantom, lung

## Abstract

Development of patient-specific CT imaging phantoms with randomly incorporated lesions of various shapes and sizes for calibrating image intensity and validating quantitative measurement software is very challenging. In this investigation, a physical phantom that accurately represents a patient's specific anatomy and the intensity of lung CT images at the voxel level will be fabricated using fused deposition modeling (FDM) 3D printing. Segmentation and modeling of a patient's CT data were performed by an expert and the results were confirmed by a thoracic radiologist with more than 20 years of experience. This facilitated the extraction of the details of the patient's anatomy; various kinds of nodules with different shapes and sizes were randomly added to the modeled lung for evaluating the size-accuracy of the quantification software. To achieve these Hounsfield Units (HU) ranges for the corresponding voxels in acquired CT scans, the infill ratios of FDM 3D printing were controlled. Based on CT scans of the 3D printed phantoms, the measured HU for normal pulmonary parenchyma, ground glass opacity (GGO), and solid nodules were determined to be within target HU ranges. The accuracy of the mean absolute difference and the mean relative difference of nodules were less than 0.55 ± 0.30 mm and 3.72 ± 1.64% (mean difference ± 95 CI), respectively. Patient-specific CT imaging phantoms were designed and manufactured using an FDM printer, which could be applied for the precise calibration of CT intensity and the validation of image quantification software.

## 1 Introduction

The manufacturing of patient-specific computed tomography (CT) imaging phantoms for calibrating CT image intensity, validating quantitative measurement software, etc, is very challenging.

Although various types of patient-specific chest phantoms have been developed, an appropriate measurement method for standard imaging phantoms that is capable of determining the measurement accuracy of software and patient-specific imaging phantoms has not been developed using 3D printing technology.^[1]^

Chest CT scans show diverse anatomic structures with a wide variety of CT intensities ranging from -1024 to 3072 Hounsfield Units (HU) including the airway, pulmonary parenchyma, diseased imaging patterns, fat, soft tissue, and bone.^[2]^ These structures can be used to identify the early onset of lung diseases such as lung cancer, emphysema, and diffuse infiltrative diseases. In addition, due to the high dependence of diagnosis on imaging, better reliability of pulmonary CT is necessary.^[3]^ In addition, the development of CT phantoms that can be used to evaluate the accuracy and reliability of pulmonary CT quantification software is also a challenge.^[4]^ Although many patient-specific chest phantoms can now be created due to recent advances in 3D printing technology, the ability to simulate sections of the phantoms is limited because of the limitations associated with the available printing materials.^[5,6]^

The purpose of this study is to simulate the shape and CT values of pulmonary parenchyma and lesions of various sizes using 3D printing technology and to verify the accuracy of CT software. Lungs, in particular, have a wide range of HU (-1024 to 400 HU) because of the presence of air, water, fat, and nodules. We investigated the HU of various 3D printing materials with various infill ratios with the goal of reproducing the HU range of chest CT images obtained from human subjects. In addition, a section of the lung from a patient's CT data was used to extract the details of their anatomy and lesions of different shapes and sizes were randomly incorporated into the modeled lung to evaluate the size-accuracy of the quantification software.

## 2 Methods

This study was approved by the Asan Medical Center Institutional Review Board with a waiver of a written informed consent to a patient due to a retrospective study without any additional harm.

To confirm the HU of a 3D printed phantom, samples with a specific size (radius 20 mm and height 30 mm) and different amounts of infill were outputted for various kinds of FDM materials including acrylonitrile butadiene styrene (ABS), thermoplastic polyurethane (TPU), and polylactic acid (PLA). For each sample, CT scans were performed^[7,8]^ to evaluate HU.

**Figure 1:**
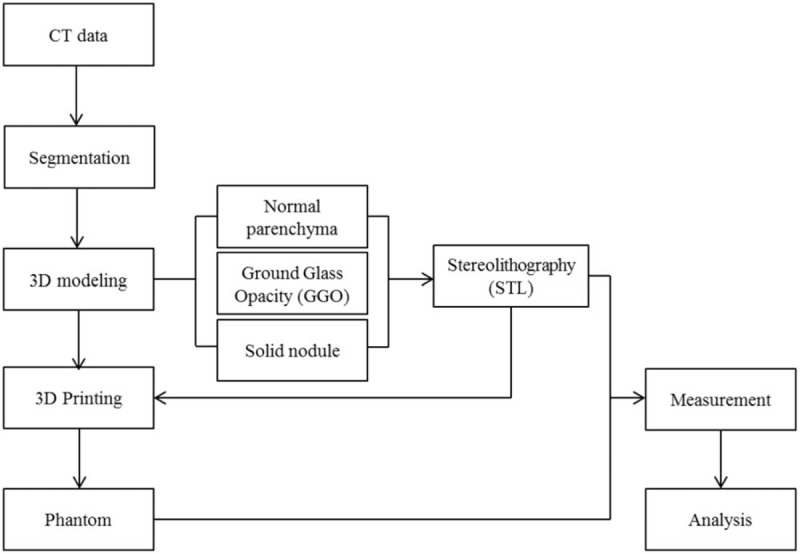
Flowchart of the procedure for producing 3D printed phantoms.

Chest CT data of a patient were acquired and segmentation and 3D modeling were performed. Data for the regions of interest (ROI) including lung parenchyma and pulmonary vessels were converted to stereolithography (STL) files. FDM 3D printing was used to fabricate phantoms for the parenchyma and lesions. Then, the final phantoms and STL files were compared (Fig. 1).

### 2.1 Subject CT scan protocols

An anonymous patient, various kinds of material samples for fabricating phantoms, and 3D printed lung phantoms were scanned using a dual-source CT (SOMATOM Definition Flash, Siemens Healthcare) with a standard protocol of 120 kVp and 1.0 mm slice thickness. In addition, these scan data were reconstructed to 0.6 mm in the axial section using software (Syngo CT 2012B).^[9]^

### 2.2 A study on 3D printing material

**Figure 2:**
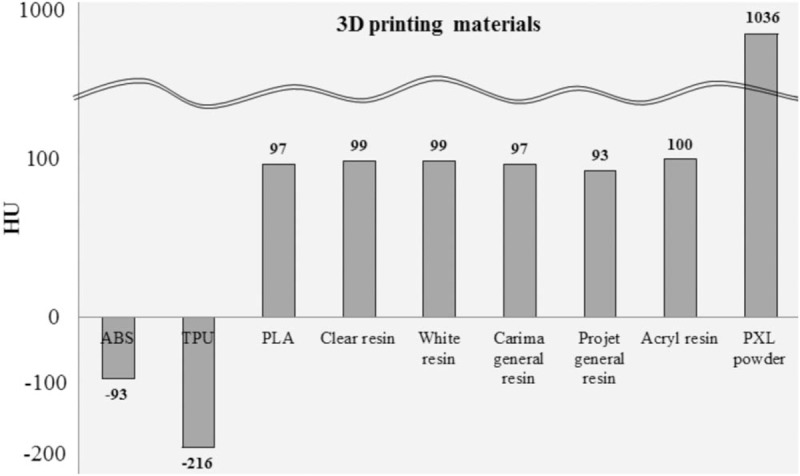
The Hounsfield Units (HU) of various 3D printing materials including acrylonitrile butadiene styrene (ABS), thermoplastic polyurethane (TPU), and polylactic acid (PLA), clear resin (Form lab.), white resin (Form lab.), general resin (Carima), general resin (Projet), Acryl resin, PXL clear powder with 100% infill ratio.

**Figure 3:**
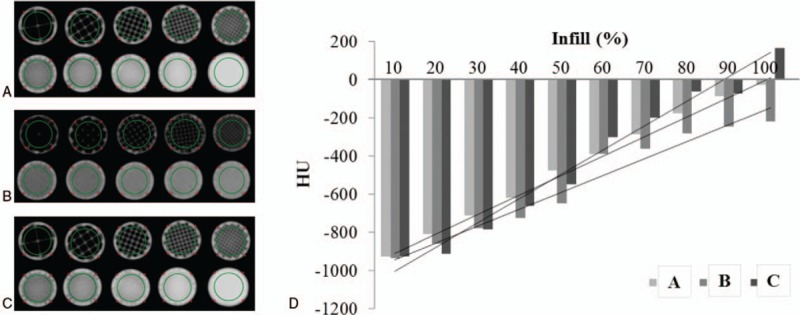
HU for the FDM materials including ABS, TPU and PLA with different amounts of infill. (A) ABS acquired CT data (B) TPU acquired CT data (C) PLA acquired CT data (D) HU for each printing material with different amounts of infill.

We investigated the applicability of various kinds of printing materials for 3D printers (Table 1) and modeled the phantoms to a specific size (20 × 50 × 5) mm^3^ using 3-matic software (Materialise Inc., Leuven, Belgium).^[10–12]^ For CT scanning, the model was fixed to a self-manufactured plate so that each model could maintain a constant alignment. The HU for each model with various kinds of materials including ABS, TPU, and PLA, clear resin (Form lab.), white resin (Form lab.), general resin (Carima), general resin (Projet), Acryl resin, PXL clear powder was then confirmed with a CT scan (Fig. 2). For evaluating the various infill ratios of FDM printers with different materials including ABS, TPU, and PLA, circular cylinders with a radius of 20 mm and a height of 30 mm (Fig. 3A–C) were designed. The percentage of the infill was controlled using Ultimaker Cura, which is a 3D printing slicer software for FDM. To determine the HU of materials with different infill ratios, a MDCT scan was also performed.

**Table 1:**
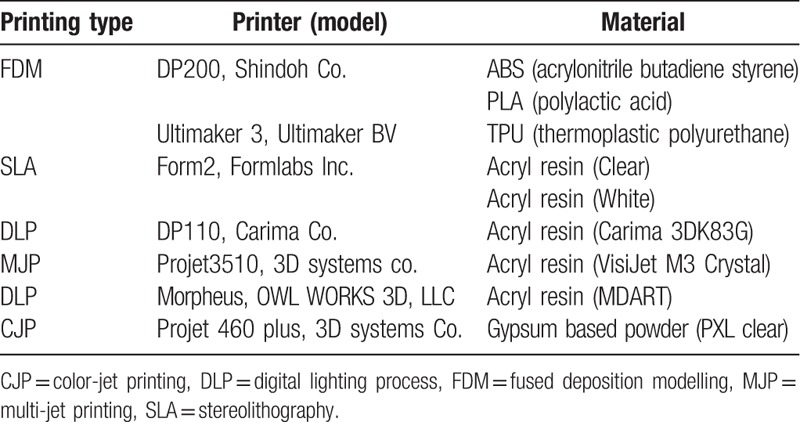
Summary of the various materials used by 3D printers.

### 2.3 The design of a patient-specific lung phantom

The right lung lobe, airway, and lesions from anonymous patient chest CT images were manually segmented using Mimics software (Materialise Inc., Louvain, Belgium). The obtained chest areas were exported to the 3-matic software (Materialise Inc., Leuven, Belgium) for post-processing. The various well-known shapes and sizes of lung diseases including ground glass opacity (GGO) and solid nodules were artificially modeled and inserted in the images. Each phantom was designed and manufactured by one type of material and printer without need of assembly. Based on the HU measurements in Figures 2 and 3, abnormality lesions including GGO and solid nodules was set with 80 and 100 percent infill ratios, respectively. The lesions were set with appropriate infill ratios using the Ultimaker Cura. The final model file was converted into an STL file format for printing using the FDM printers.

### 2.4 Statistical evaluation

To evaluate the HU range and to compare the sizes of the designed STL model and the actual model, the 3D printed phantom was scanned using the same dual-source CT equipment and scan protocol used for the initial study. Several ROIs of the designed STL model including normal parenchyma, GGO, and solid nodules were selected for size measurements. In CT images of the 3D printed phantom, the HU and size of each corresponding ROI were measured five times using a RadiAnt DICOM viewer (Medixant Inc., Poznan, Poland). The measurement error of HU between reference and scanned CT values of GGO and solid nodule were evaluated by applying median and lower bound values as −350 and −100 HU, respectively. The absolute value means, and standard deviation were listed in Table 3. The size measurement results were then analyzed using a Bland-Altman method.^[13,14]^

## 3 Results

### 3.1 Baseline HU evaluation with different kinds of 3D printing materials

We evaluated the HU from sample CT images for various types of 3D printers and materials at 100 percent infill ratio (Fig. 2). These wide ranges of HU for the different types of printers and materials could be used to develop a lung phantom with different values of internal infill ratios, which could result in lower HU phantom values than the aforementioned at 100 percent infill ratio.

### 3.2 HU evaluation with different infill ratios of ABS, TPU, and PLA materials

CT scans were obtained for the ABS, TPU, and PLA samples with various amounts of infill ratios from 10% to 100% in 10% increments. The average HU for the same size ROI along the axial section of each sample were evaluated. 3D printed models with lower infill ratios have higher porosity and gradually less HU (Fig. 3).

### 3.3 Patient-specific lung imaging phantom for CT

**Figure 4:**
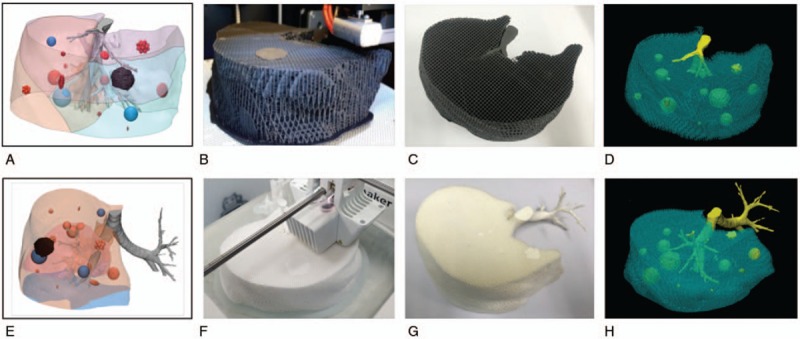
Fabrication of lung phantom using a single 3D printing technology. (A), (E) represent 3D modeling of the lung CT phantom. (B), (F) are fabricated CT phantoms using 3D printing. (C), (G) are printed final CT phantoms. (D), (H) are volume-rendered images of 3D printed CT phantoms.

**Figure 5:**
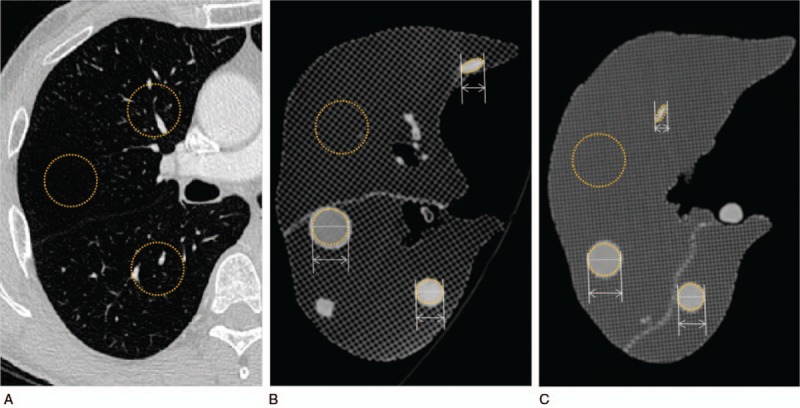
Based on the patient's CT data, the parenchyma and shape of the lung lesion were reproduced. The HU and size of each modeled lesion were measured. (A) measured CT value for the region of interest (ROI) of lung parenchyma from patient data. (B) measured CT value and length of lesions from modeled ABS phantom data. (C) measured CT value and length of lesions from modeled TPU phantom data.

Based on the aforementioned results on the infill ratios of materials,

To fabricate a patient-specific lung imaging phantom, a section in the right lobe of the lung from the patient CT images was selected to model the lung lobe, fissure, and airway. The lung phantom was fabricated using a single printer and material by controlling the HU using different amounts of infill ratios (Fig. 4). The HU of lung parenchyma and various lesions in the CT scan of the phantom were analyzed (Fig. 5). The HU for normal pulmonary parenchyma, which is mainly composed of air, is between −600 and −900 and the ABS and TPU phantom had HU that were similar to those of human patients. In addition, for a pattern of typical lung disease, GGO achieved HU that were similar to the actual values −300 to −400 (Tables 2 and 3).

**Table 2:**
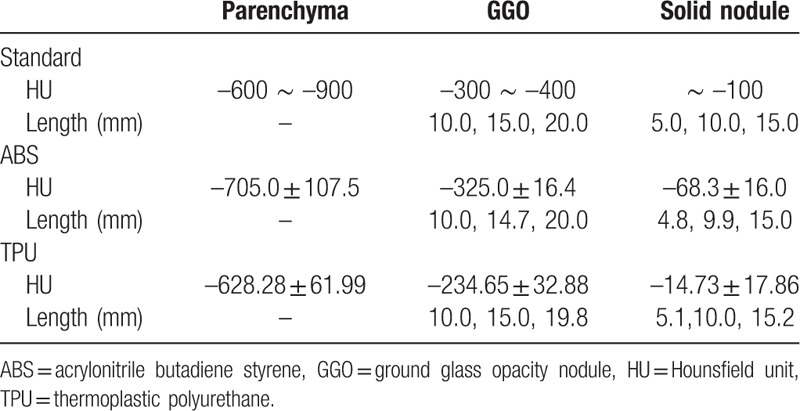
Comparison of CT-based 3D modeling measurements with software analysis of printouts.

**Table 3:**
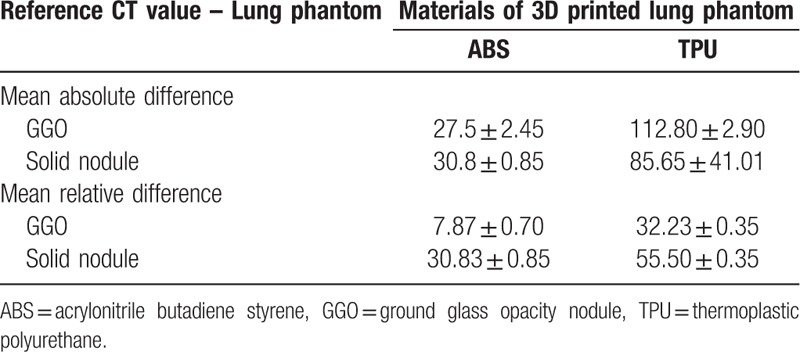
Comparison of the reference CT value and 2 types of materials of 3D printed lung phantoms.

**Figure 6:**
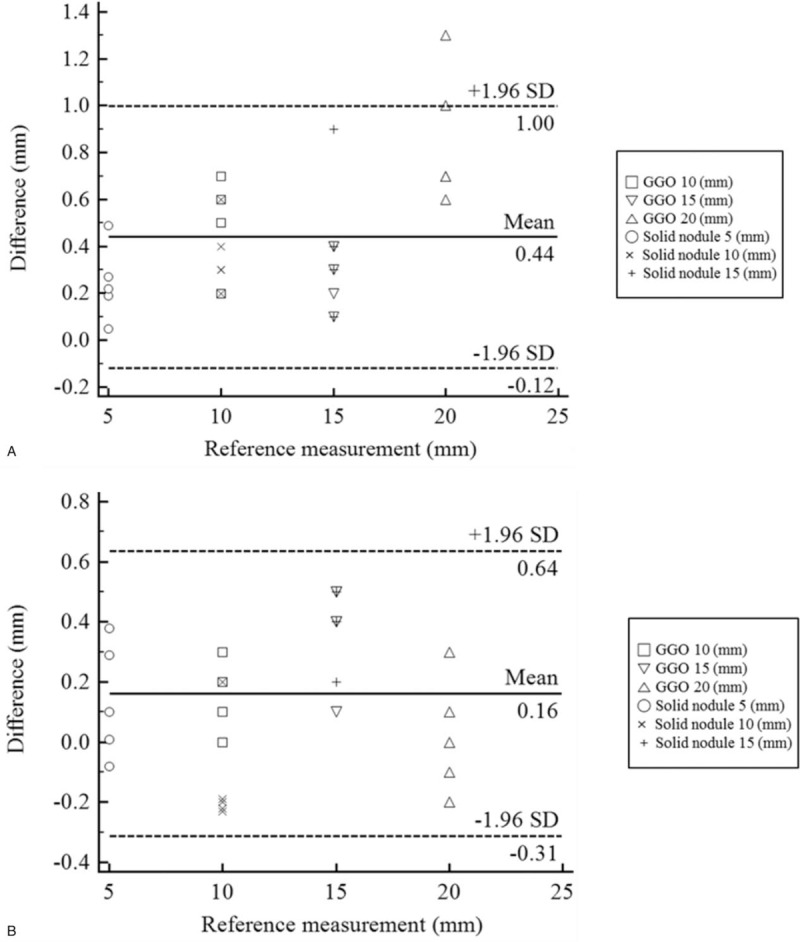
Each lesion was measured a total of 5 times (GGO, solid nodule) for the 3D phantom models and CT data (reference measurement) for the final printed phantom. (A) measured value of each disease model in the ABS phantom. (B) measured value of each disease model in the TPU phantom.

The size measurement of each lesion was evaluated using the Bland–Altman analysis. The measurement accuracies of the phantoms were examined and compared depending on the type of 3D printing material. The accuracies (mean difference ± 95 CI) of ABS and TPU are GGO: 0.55 ± 0.30 mm, solid nodule: 0.33 ± 0.08 mm and GGO: 0.19 ± 0.18 mm, solid nodule: 0.22 ± 0.21 mm, respectively (Fig. 6 and Table 4).

**Table 4:**
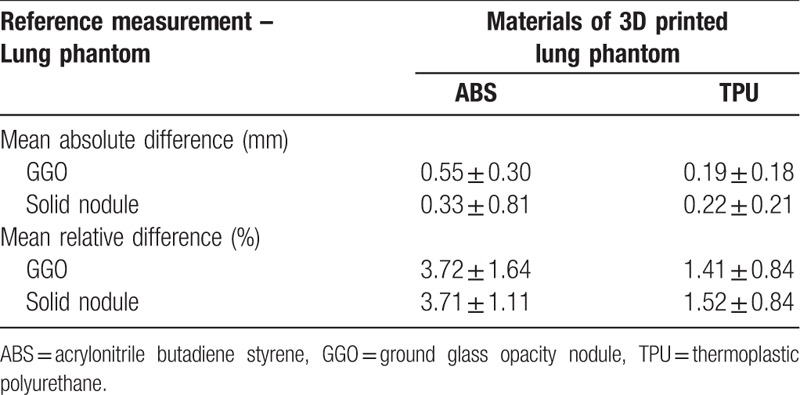
Comparison of the reference measurement and 2 types of materials of 3D printed lung phantoms.

## 4 Discussion

Up to now, phantoms are primarily used for the evaluation and maintenance of medical devices.^[15]^ However, there are several notable studies based on the application of 3D printing for the fabrication of CT imaging phantoms. For example, Hernandez-Giron et al used multi-jet 3D printing technology to fabricate an anthropomorphic lung phantom.^[16]^ However, this method is very expensive. Zhang et al, fabricated a lung phantom using 3D printing technology and tissue equivalent materials.^[17]^ This phantom has a realistic human anatomy and radiation attenuation property. Abdullah et al developed an organ-specific insert phantom using a 3D printer to investigate cardiac CT protocols. This phantom exhibited human-like chest and Hounsfield Unit (HU) characteristics that were comparable to that of a human subject, but the cardiac structure was non-anthropomorphic.^[18]^

On the other hand, in this study, we developed patient-specific CT imaging phantoms with randomly incorporated lesions with various shapes and sizes for calibrating image intensity and validating quantitative measurement software. This physical phantom was scanned using CT to evaluate HU using different materials including ABS, TPU, and PLA for FDM 3D printing, which yields a range of HU between 0 and 200. To overcome this limitation, we attempted to achieve a lower HU range of −1000 to 0 HU by controlling the infill ratios, which can be one of the setting values of 3D printing via FDM. The emphysema, normal parenchyma, and nodules have HUs of less than −950, −800 to −600, and more than −200, respectively. The measured HU for normal pulmonary parenchyma, GGO, and solid nodules were within target HU ranges of a normal chest CT. The error of the measurement accuracy was determined to be submillimeter using quantitative measurement software.

The advantage of the method proposed in this paper is that it would implement realistic morphology, and it shows that the application of 3d printing technology in various medical fields could help overcome the limits of current physical phantom by making imaging phantom. In line with our study, several 3D printed imaging phantoms have been developed with different methods, for example, Altermatt et al, by using agar gel doped with contrast agent polyvinyl chloride (PVC) and 3D printing^[19]^ or Yaoyao et al, using PVC and 3D printing.^[20]^ Additionally, Aldosari et al, produced a 3D-printed pulmonary artery model for simulating peripheral pulmonary embolism and confirmed that the shape is very similar to the patient anatomy.^[21,22]^ However, this model was limited in the choice of materials for the pulmonary embolism simulation and used an anonymous elastoplastic material with flexible features. Therefore, the CT imaging phantom was produced but did not reflect the HU well. To overcome the limitation, they proposed soaking the phantom to the contrast agent.^[23]^ Unlike previous other studies, we could use only one FDM printer to fabricate the lung imaging phantom without assembly. This 3D printing technology was able to implement a wide range of HU. Moreover, FDM printers are the most economical 3D printers, which is advantageous for future clinical application.

There could be various kinds of clinical applications of this patient-specific imaging phantom. One important application is the validation of quantification software. Various kinds of measurements on airway dimension and tumor diameter using different software with the various algorithms are well-known issues need to be overcome.^[24,25]^ The performance of software in difficult setting such as a complex anatomic lesion, that is branching area of the airway, cannot be evaluated corrected using the commercial phantom with the typical anatomic model. Generating the phantom with similar complex anatomy of the human body and similar CT density profile would be useful to validate software because the real dimension of the physical phantom can be controlled easily. The second potential application is for the education, having realistic physical phantom along with CT images would enhance the efficiency of training image interpretation skills to medical students and radiology residents. Finally, this patient-specific model will be useful to the clinician to communicate with the patient explaining the specific disease condition and discussing the treatment plan and so on.

There are several limitations in our current prototype phantom study. The proposed method has a limitation to print bone structures. Most commercially available chest phantoms include the chest wall and bones such as ribs and the spine. To incorporate spine and bony structures using 3D printing, the CT value of bone can be replicated using color-jet printing (CJP) materials, which have HUs of 1000 or more, by assembling with an FDM lung model. For further investigation, a higher range up to +1000 HU can be obtained using contrast agents with different concentrations by injecting them into voids inside 3D printed parts.^[26]^ The other limitation includes that the texture of the lung parenchyma is unnatural. CT image of the phantom showed the regular infill pattern of walls in the parenchyma, which are characteristic of FDM 3D printing. It is very difficult to remove these walls. We think that this limitation can be overcome by using the characteristics, which were generally regarded as disadvantages of FDM technology.^[27,28]^ FDM 3D printing is a method of squeezing filaments made of polymer material from a printing head, and laminating layer by layer. As the temperature of the printing head is high and the moving speed is slow, string-like structures appear on the surface.^[29]^ This could be used to implement the form of alveoli. Another method is to use foamed silicone, where the degree of foaming depends on negative pressure.^[30,31]^

In summary, the CT values of different 3D printed materials were evaluated and CT imaging lung phantoms were fabricated using FDM 3D printing based on a patient's CT data, to include anatomical details. The generation of patient-specific lung imaging phantom using only 3D printing is difficult. However, this study demonstrates that a wide range of CT values can be achieved by controlling the internal filling of the 3D printing material. In addition, a variety of lesions of different shapes and sizes were randomly incorporated into the lung phantom to evaluate the size-accuracy of quantification software.

## Acknowledgments

We would like to thank Younghwa Byeon for his excellent technical assistance and English editing.

## Author contributions

**Conceptualization:** Sangwook Lee, Namkug Kim, Joon Beom Seo.

**Data curation:** Dayeong Hong.

**Investigation:** Dayeong Hong.

**Methodology:** Dayeong Hong, Sangwook Lee, Guk Bae Kim, Namkug Kim.

**Project administration:** Dayeong Hong, Namkug Kim.

**Resources:** Namkug Kim.

**Software:** Dayeong Hong, Sangwook Lee.

**Supervision:** Guk Bae Kim, Sang Min Lee, Namkug Kim, Joon Beom Seo.

**Validation:** Dayeong Hong, Sang Min Lee, Namkug Kim, Joon Beom Seo.

**Visualization:** Dayeong Hong.

**Writing – original draft:** Dayeong Hong, Namkug Kim.

**Writing – review & editing:** Dayeong Hong, Guk Bae Kim, Namkug Kim, Joon Beom Seo.

Dayeong Hong orcid: 0000-0002-6270-9643.
